# The Urinary Glucose Excretion by Sodium–Glucose Cotransporter 2 Inhibitor in Patients With Different Levels of Renal Function: A Systematic Review and Meta-Analysis

**DOI:** 10.3389/fendo.2021.814074

**Published:** 2022-01-27

**Authors:** Suiyuan Hu, Chu Lin, Xiaoling Cai, Xingyun Zhu, Fang Lv, Lin Nie, Linong Ji

**Affiliations:** ^1^Department of Endocrinology and Metabolism, Peking University People’s Hospital, Beijing, China; ^2^Department of Endocrinology and Metabolism, Beijing Airport Hospital, Beijing, China

**Keywords:** urinary glucose excretion, estimated glomerular alteration rate (eGFR), creatine clearance, renal function impairment, sodium–glucose cotransporter 2 (SGLT2) inhibitor

## Abstract

**Objective:**

Previous evidence suggested that sodium–glucose cotransporter 2 inhibitor (SGLT2i)-mediated urinary glucose excretion (UGE) appeared to be reduced with a decrease in glomerular filtration rate. Thus, we conducted a systematic review and meta‐analysis to compare SGLT2i-mediated UGE among individuals with different levels of renal function.

**Methods:**

We conducted systematic searches in PubMed, Medline, Embase, Cochrane Central Register of Controlled Trials, and ClinicalTrial.gov from inception to May 2021. Clinical studies of SGLT2i with reports of UGE changes in predefined different levels of renal function were included. The results were expressed as pooled effect sizes with 95% confidence interval (CI). A random-effects model was used to calculate the pooled effect sizes.

**Results:**

In total, eight eligible studies were included. Significant differences were observed in the post-treatment UGE level among subgroups stratified by renal function (*P <*0.001 for subgroup difference), which were gradually decreased along with the severity of impaired renal function. Consistently, changes in UGE before and after SGLT2i treatment were also decreased along with the severity of impaired renal function [67.52 g/day (95%CI: 55.58 to 79.47 g/day) for individuals with normal renal function, 52.41 g/day (95%CI: 38.83 to 65.99 g/day) for individuals with mild renal function impairment, 35.11 g/day (95%CI: 19.79 to 50.43 g/day) for individuals with moderate renal function impairment, and 13.53 g/day (95%CI: 7.20 to 19.86 g/day) for individuals with severe renal function impairment; *P <*0.001 for subgroup differences].

**Conclusions:**

SGLT2i-mediated UGE was renal function dependent, which was decreased with the extent of renal function impairment.

## Introduction

Sodium–glucose cotransporter 2 inhibitors (SGLT2i) are widely used for the treatment of type 2 diabetes mellitus (T2DM) (1, 2). SGLT2i increases urinary glucose excretion (UGE) by blocking the reabsorption of glucose in the renal proximal tubule (3). Thus, SGLT2i-mediated glycosuria is associated with improved glycemic control and reduced levels of glycosylated hemoglobin (HbA1c) (4). Since SGLT2i exerted their effects in the kidneys, it was advised to make specific dose adjustments based on renal function when giving a prescription (5). Moreover, studies have shown that the glucose-lowering effect of SGLT2i was dependent on glomerular filtration (6). Compared to patients with normal renal function or mild chronic kidney disease (CKD), the hypoglycemic efficacy of SGLT2i was reduced in patients with moderate CKD (7).

Therefore, it is reasonable to assume that UGE induced by SGLT2i might decline with worsening renal impairment [as indicated by a reduction in estimated glomerular filtration rate (eGFR)]. Although it was reported that the magnitudes of UGE were associated with eGFR in certain pharmacokinetic and pharmacodynamic studies of SGLT2i, a systematic evaluation with synthesized data seemed to be absent. To assess the changing tendency of SGLT2i-medicated UGE along different levels of renal function, we conducted a systematic review and meta-analysis to compare the differences in UGE after treatment of SGLT2i among patients with different levels of renal function.

## Materials and Methods

### Data Sources and Search Strategy

The systematic review and meta‐analysis were reported according to the Preferred Reporting Items for Systematic Reviews and Meta-analyses guidelines. This meta-analysis was registered in PROSPERO platform as CRD42021277970. We searched PubMed, Medline, Embase, Cochrane Central Register of Controlled Trials, and ClinicalTrial.gov databases from inception to May 2021. Several medical subject headings as well as free-text search terms were used in our searches, including SGLT2 inhibitors, canagliflozin, dapagliflozin, empagliflozin, ertugliflozin, ipragliflozin, luseogliflozin, remogliflozin, sotagliflozin, tofogliflozin, clinical trials, pharmacokinetics, pharmacodynamics, renal dysfunction, renal impairment, and chronic kidney disease. Related references in existing reviews in this field were also screened to identify additional relevant studies.

Two reviewers (CL and SH) respectively browsed the titles, abstracts, and full texts of potentially eligible studies. The candidate studies were then re-examined for inclusion according to predefined criteria. Any disagreements were resolved by consultation with a third reviewer (XZ).

### Inclusion and Exclusion Criteria

The inclusion criteria were as follows: (a) clinical trials of SGLT2i, (b) clinical trials conducted in individuals with different extent of renal dysfunction that reported changes in 24-h UGE in predefined different levels of renal function, (c) manuscripts published in English, and (d) participants who were at least 18 years old. Studies were excluded if they met the following criteria: (a) the trials were animal studies, (b) clinical trials of non-SGLT2i, (c) clinical trials that did not report UGE data in predefined different levels of renal function, and (d) clinical trials that were unable to extract the absolute data of UGE.

### Data Extraction and Quality Assessment

Two reviewers (CL and SH) used a standard form to extract the following properties of each included study: study characteristics (first author, publication year, and study design), participant characteristics (sample size, age, sex, disease type, and baseline 24-h UGE), therapeutic intervention (type of SGLT2i, dose, and frequency and duration of treatment), outcomes of interest [post-treatment UGE level (24 h) as well as means and standard deviations or standard errors of changes in UGE (24 h) in the treatment groups]. Renal function stratifications were predefined in this meta-analysis as follows: normal renal function, eGFR ≥90 ml/min/1.73 m^2^ or creatine clearance ≥80 ml/min; mild renal function impairment, eGFR 60 to <90 ml/min/1.73 m^2^ or creatine clearance 50 to <80 ml/min; moderate renal function impairment, eGFR 30 to <60 ml/min/1.73 m^2^ or creatine clearance 30 to <50 ml/min; and severe renal function impairment, eGFR <30 ml/min/1.73 m^2^ or creatine clearance <30 ml/min.

Two authors (CL and SH) independently evaluated the quality of the included studies using the Cochrane risk-of-bias tool. Publication bias was assessed *via* funnel plots. Any disagreements were resolved by consensus with a third author (XZ).

### Data Synthesis and Statistical Analysis

The outcome measures in our meta-analysis were pooled effect sizes of post-treatment UGE level (24 h) or changes in UGE (24 h) (calculated as differences between post-treatment value and baseline value) and their 95%CI values. The degree of between‐study heterogeneity was calculated using the Higgins *I*^2^ statistics. Fixed-effects model was used when *I*^2^ <50%, and random-effects model was used when *I*^2^ ≥50%. ANOVA tendency analysis was conducted to test the association between UGE and renal function. The meta-analyses were primarily performed by the Review Manager statistical package (version 5.3, Nordic Cochrane Centre, Copenhagen, Denmark). The ANOVA tendency analysis was conducted by SPSS software (SPSS 24.0. Armonk, NY: IBM Corp). Statistical significance was considered at *P <*0.05.

## Results

### Study Selection and Characteristics

**Figure 1:**
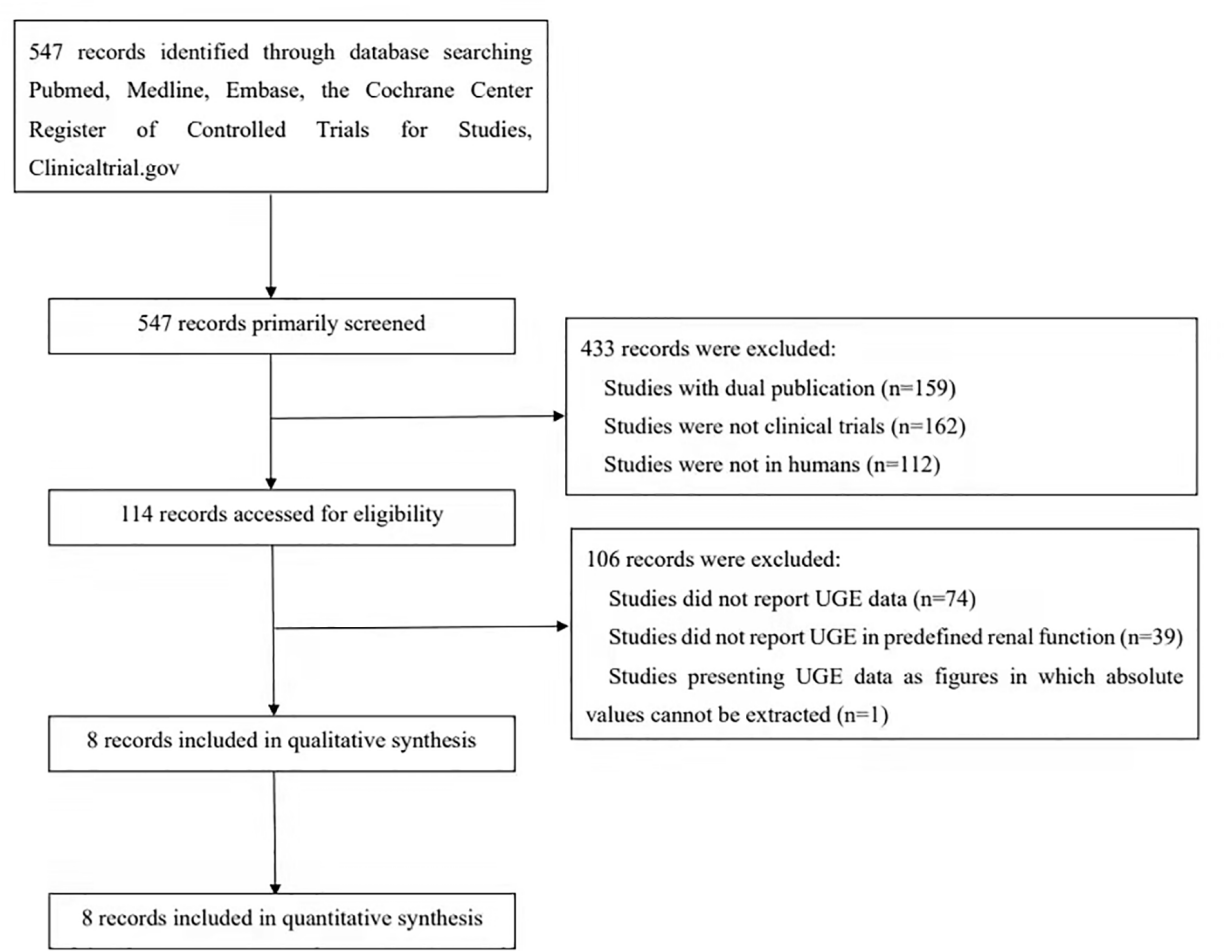
Flow chart of the included studies.

The process of study search and selection is shown in **Figure 1**. This led to an inclusion of 8 studies with 274 participants in our meta-analysis. SGLT2i, including canagliflozin, empagliflozin, ertugliflozin, tofogliflozin, luseogliflozin, and ipragliflozin, was assessed. The number of patients with normal renal function, mild renal impairment, moderate renal impairment, and severe renal impairment was 68, 75, 79, and 52, respectively. The study conducted by Kasichayanula et al. demonstrates the UGE change from baseline in patients with renal dysfunction after the administration of dapagliflozin. Unfortunately, the results were illustrated in supplements as histograms, and we were unable to extract the exact values. The baseline characteristics of the included studies are summarized in **Table 1**. The Cochrane risk-of-bias tool indicated the concerns of bias arising from inadequate randomization and blinding (**Supplementary Table S1**). The funnel plots also showed uneven distributions, which indicated potential publication bias (**Supplementary Figures S1**, **S2**).

**Table 1:** Baseline characteristics for studies of urinary glucose excretion assessment in patients with renal dysfunction.

Study (author, year)	Treatment	Renal dysfunction group	Definition of renal dysfunction	Number of participants
Devineni et al., 2015 (2)	Canagliflozin, 200 mg	Normal renal function	Creatine clearance, ≥80 ml/min	8
		Mild renal impairment	Creatine clearance, 50 to <80 ml/min	8
		Moderate renal impairment	Creatine clearance, 30 to <50 ml/min	8
		Severe renal impairment	Creatine clearance, <30 ml/min	8
Ikeda et al., 2019 (3)	Tofogliflozin, 20 mg	Normal renal function	eGFR^a^, >80 ml/min/1.73 m^2^	11
		Mild renal impairment	eGFR^a^, 50 to ≤80 ml/min/1.73 m^2^	8
		Moderate renal impairment	eGFR^a^, 30 to <50 ml/min/1.73 m^2^	9
		Severe renal impairment	eGFR^a^, <30 ml/min/1.73 m^2^	8
Macha et al., 2015 (4)	Empagliflozin, 50 mg	Normal renal function	eGFR, ≥90 ml/min/1.73 m^2^	8
		Mild renal impairment	eGFR, 60 to <90 ml/min/1.73 m^2^	9
		Moderate renal impairment	eGFR, 30 to <60 ml/min/1.73 m^2^	7
		Severe renal impairment	eGFR, <30 ml/min/1.73 m^2^	8
Sahasrabudhe et al., 2017 (5)	Ertugliflozin, 15 mg	Normal renal function	eGFR, ≥90 ml/min/1.73 m^2^	6
		Mild renal impairment	eGFR, 60 to <90 ml/min/1.73 m^2^	8
		Moderate renal impairment	eGFR, 30 to <60 ml/min/1.73 m^2^	8
		Severe renal impairment	eGFR, <30 ml/min/1.73 m^2^	6
Samukawa et al., 2018 (6)	Luseogliflozin, 5 mg	Normal renal function	eGFR, ≥90 ml/min/1.73 m^2^	11
		Mild renal impairment	eGFR, 60 to <90 ml/min/1.73 m^2^	17
		Moderate renal impairment G3a	eGFR, 45 to <60 ml/min/1.73 m^2^	10
		Moderate renal impairment G3b	eGFR, 30 to <45 ml/min/1.73 m^2^	13
		Severe renal impairment	eGFR, <30 ml/min/1.73 m^2^	6
Sarashina et al., 2014 (7)	Empagliflozin, 25 mg	Normal renal function	eGFR, ≥90 ml/min/1.73 m^2^	8
		Mild renal impairment	eGFR, 60 to <90 ml/min/1.73 m^2^	8
		Moderate renal impairment	eGFR, 30 to <60 ml/min/1.73 m^2^	8
		Severe renal impairment	eGFR, <30 ml/min/1.73 m^2^	8
Smulders et al., 2011 (8, 9)	Ipragliflozin, 50 mg	Normal renal function	eGFR, ≥90 ml/min/1.73 m^2^	8
		Mild renal impairment	eGFR, 60 to <90 ml/min/1.73 m^2^	9
		Moderate renal impairment	eGFR, 30 to <60 ml/min/1.73 m^2^	8
Veltkamp et al., 2011 (9, 10)	Ipragliflozin, 100 mg	Normal renal function	eGFR, ≥90 ml/min/1.73 m^2^	8
		Mild renal impairment	eGFR, 60 to <90 ml/min/1.73 m^2^	8
		Moderate renal impairment	eGFR, 30 to <60 ml/min/1.73 m^2^	8
		Severe renal impairment	eGFR, <30 ml/min/1.73 m^2^	8

eGFR, estimated glomerular filtration rate.

a Since the renal function stratifications in this study basically matched with what we predefined, the data were also included in our analysis.

In our analyses, we adopted three different kinds of grouping to make between-group comparisons. One is what we pre-mentioned in “Methods”, in which patients were divided into four groups according to eGFR or creatine clearance levels, including normal renal function, mild renal function impairment, moderate renal function impairment, and severe renal function impairment. Besides this, we divided the participants into two groups: those taking eGFR at 60 or 30 ml/min/1.73 m^2^ as the cutoff value, respectively. Our results showed that, with deterioration of renal function, the absolute UGE levels and SGLT2i-related UGE elevation declined progressively.

### Effects of SGLT2i on Post-treatment Absolute UGE Levels in Patients With Different Levels of Renal Function

**Figure 2:**
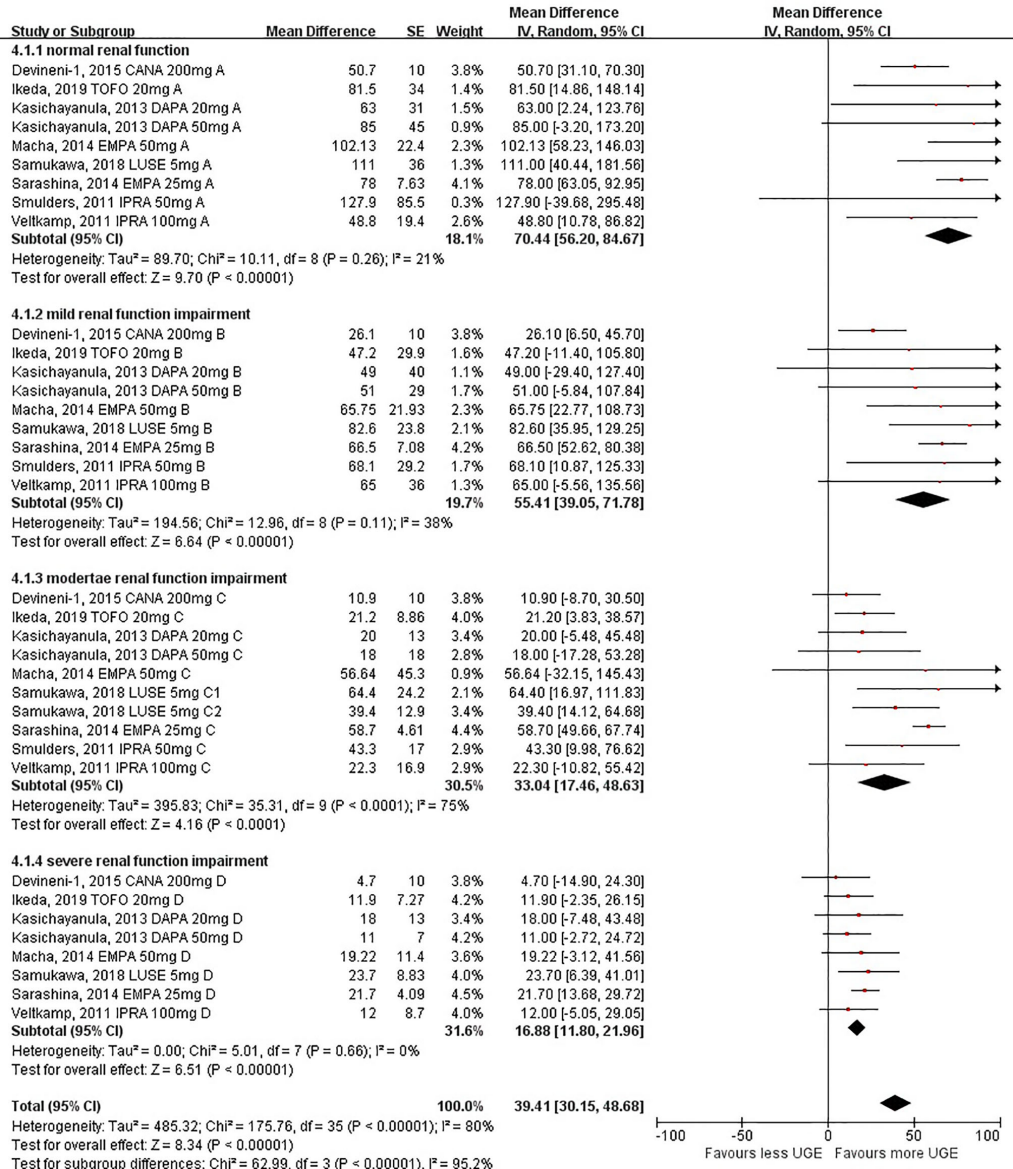
Post-treatment urinary glucose excretion in patients with different levels of renal function.

In the pooled analysis of 8 studies reporting post-treatment absolute UGE levels, the results showed that the post-treatment UGE was lower in patients with worse renal impairment. Compared with patients with normal renal function, the post-treatment UGE started to decline in the subgroup with mild renal function impairment (55.41 g/day, 95%CI: 39.05 to 71.78 g/day), while a more prominent decrease of UGE was observed in the subgroup with moderate renal function impairment (33.04 g/day, 95%CI: 17.46 to 48.63 g/day). The UGE in the subgroup with severe renal function impairment (16.88 g/day, 95%CI: 11.80 to 21.96 g/day) diminished drastically (**Figure 2**). A linear declining association between post-treatment UGE and levels of renal function was found by tendency analysis (linear term, *F* = 59.89, *P* < 0.001) (**Supplementary Table S3**).

The subgroup analyses further confirmed the significant decrease in post-treatment UGE for individuals with substantial renal function impairment [62.93 g/day (95%CI: 51.66 to 74.21 g/day) for individuals with eGFR ≥60 ml/min/1.73 m^2^ and 23.67 g/day (95%CI: 14.17 to 33.16 g/day) for individuals with eGFR <60 ml/min/1.73 m^2^ (**Supplementary Figure S5**); *P* < 0.001 for subgroup differences]. When eGFR at 30 ml/min/1.73 m^2^ was taken as the cutoff value, the difference between the two groups remained significant [49.93 g/day (95%CI: 39.73 to 60.13 g/day) for individuals with eGFR ≥30 ml/min/1.73 m^2^ and 16.88 g/day (95%CI: 11.80 to 21.96 g/day) for individuals with eGFR <30 ml/min/1.73 m^2^; P < 0.001 for subgroup differences] (**Supplementary Figure S6**).

### Effects of SGLT2i on UGE Changes From Baseline in Patients With Different Levels of Renal Function

**Figure 3:**
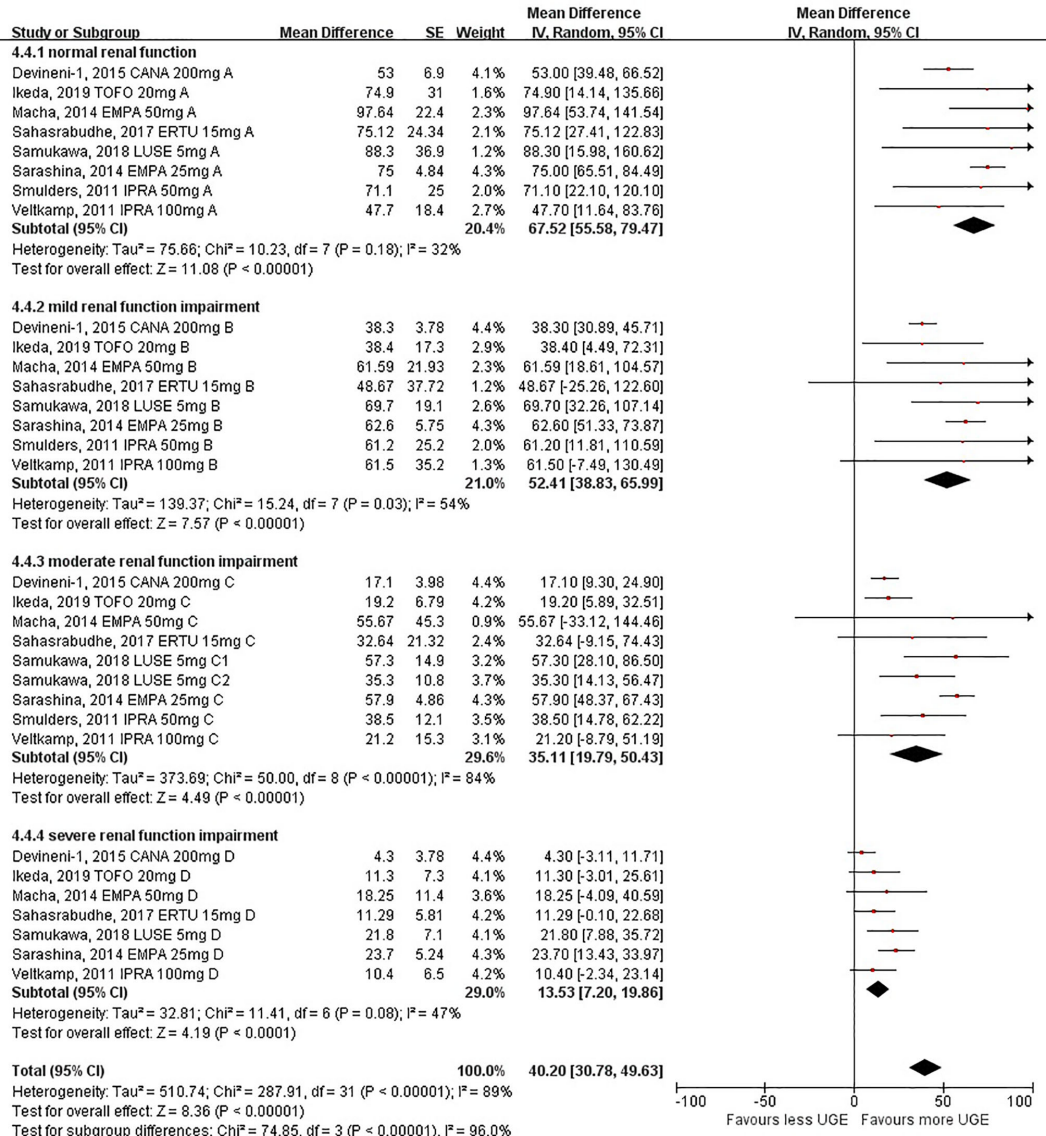
Urinary glucose excretion changes before and after sodium–glucose cotransporter 2 inhibitor treatment in patients with different levels of renal function.

UGE changes from the baseline (calculated as differences between post-treatment UGE values and baseline UGE values) followed the same pattern as observed in post-treatment absolute UGE among different levels of renal function, indicating that SGLT2i-mediated UGE seemed to be weakened when the renal function got worse [67.52 g/day (95%CI: 55.58 to 79.47 g/day) for individuals with normal renal function, 52.41 g/day (95%CI: 38.83 to 65.99 g/day) for individuals with mild renal function impairment, 35.11 g/day (95%CI: 19.79 to 50.43 g/day) for individuals with moderate renal function impairment, and 13.53 g/day (95%CI: 7.20 to 19.86 g/day) for individuals with severe renal function impairment; *P <*0.001 for subgroup differences] (**Figure 3**). The ANOVA tendency analysis indicated a linear declining association between UGE changes and levels of renal function (linear term, *F* = 73.65, *P* < 0.001) (**Supplementary Table S5**).

Similarly, when eGFR at 60 or 30 ml/min/1.73 m^2^ was taken as the cutoff value, respectively, a significant decrease in UGE changes was also observed in individuals with substantial renal function impairment [60.60 g/day (95%CI: 49.39 to 71.81 g/day) for individuals with eGFR ≥60 ml/min/1.73 m^2^ and 24.21 g/day (95%CI: 14.96 to 33.46 g/day) for individuals with eGFR <60 ml/min/1.73 m^2^ (**Supplementary Figure S7**); *P <*0.001 for subgroup differences; 50.28 g/day (95%CI: 40.08 to 60.47 g/day) for individuals with eGFR ≥30 ml/min/1.73 m^2^ and 13.53 g/day (95%CI: 7.20 to 19.86 g/day) for individuals with eGFR <30 ml/min/1.73 m^2^; *P <*0.001 for subgroup differences] (**Supplementary Figure S8**).

## Discussion

Combined with the available evidence from clinical trials, we found that, with SGLT2i use, there was a significant difference in the absolute value of post-treatment UGE among patients with different levels of renal function. The absolute value of post-treatment UGE gradually decreased with the worsening of renal function. Similarly, for UGE changes from baseline after the use of SGLT2i, a significant difference was also found among patients with different levels of renal function. The UGE changes from baseline also gradually decline with the worsening of renal function.

Reduced UGE in patients with renal dysfunction was observed among different SGLT2i subtypes. It was reported that renal glucose clearance was reduced by 42, 83, and 84% after treatment with dapagliflozin in patients with mild, moderate, or severe renal impairment, respectively (8). Another study indicated that the 24-h UGE after canagliflozin administration decreased as renal function declined in patients with T2DM. The elevation of 24-h UGE from baseline in patients with moderate CKD was only 70% of that in patients with normal renal function or mild CKD (9). Similarly, following treatment with empagliflozin and ipragliflozin, the cumulative UGE over 24 h decreased with the increasing severity of renal impairment and was correlated with eGFR (10–12). It was found that the plasma concentrations of SGLT2i were progressively increased with declining renal function, while a greater systemic exposure did not lead to a corresponding increase in renal glucose clearance (6). As shown in a previous study, through direct glucuronide conjugation, dapagliflozin was metabolized to dapagliflozin 3-O-glucuronide (D3OG) (13), which failed to show a meaningful inhibition of SGLT2 at clinically relevant doses. What is more, the extent of the increase in steady-state Cmax (maximum observed plasma concentration) for D3OG was much higher than dapagliflozin when the renal impairments got more severe (8).

Although UGE decreased with declining eGFR, it was found that the inhibition towards renal glucose reabsorption was constant across all renal function groups (14). The fractional glucose excretion of ipragliflozin was likewise maintained in patients with severe CKD (12). Such findings indicated that, regardless of renal function, inhibition of glucose reabsorption might reach its maximum. Similarly, data showed that a 20-mg dose of dapagliflozin resulted in the plateau of the exposure–response relationship in subjects with CLCR ≤50 ml min^−1^ (15, 16).

It was speculated that the action of additional SGLT2 or other tubular transport mechanisms, such as SGLT1, limited the complete inhibition in filtered glucose of SGLT2i. Alternatively, the increased tubular concentration of glucose (greater filtered glucose load) seemed to compete with SGLT2i from binding to SGLT2 and thus might suppress the degree of glucose reabsorption inhibition (10).

It was also supposed that the decrease in UGE followed the same pattern as the glucose-lowering effect progressively attenuated with impaired renal function (measured by HbA1c) (17), although in rodent models of diabetes, it was suggested that glycosuria may not be the only mechanism for the glucose-lowering effect of SGLT2 inhibitors. A previous study indicated that dapagliflozin seemed to improve glycemic control, in part through inhibiting hepatic glucagon signaling (18). Our results showed a descending tendency of SGLT2i-mediated urinary glucose loss with the decline of renal function. Therefore, this was in accordance with the recommendation to assess the renal function before initiating treatment with SGLT2 inhibitors since the balance between hypoglycemic efficacy and adverse effects is disrupted as renal function declines.

By far, several large randomized cardiovascular outcome trials suggested that SGLT2i may exert cardiovascular- and renal-protective effects even in patients with renal impairment (19–22). Recent meta-analyses have indicated that cardiovascular and renal benefits were well preserved in patients with substantial renal function impairment, suggesting that SGLT2i potentially plays a sustained role in patients with limited eGFR and diminished UGE (23). Thus, more extensive clinical studies are required to evaluate the possible relationship between SGLT2i-mediated UGE and cardiovascular and renal benefits.

## Limitations

Our meta-analysis also has several limitations. First, although we did our best to collect all available data, the specific value reflecting UGE was rarely reported. Thus, the clinical trials included in our analysis were limited, and the sample size was insufficient. We could only establish a relative trend, but not a quantized value. This also limits us from performing a further subgroup analysis towards drug types. Moreover, assessments towards risk of bias suggested that our included studies were at a certain risk of selection bias and performance, and the funnel plots showed a potential publication bias. Thus, this might compromise the reliability of the results, which should be interpreted with caution. In addition, other factors, such as baseline blood glucose level or concomitant medication that may influence UGE, were not evaluated, which may serve as confounding factors in the analyses. Moreover, the classification of renal function in this meta-analysis was defined according to the eGFR value. There was no available data about the urinary albumin excretion rate (UACR) level in the included studies among patients with different extent of renal impairment. Therefore, we were unable to analyze the associations between the UGE changes and UACR levels in patients with different extent of renal dysfunction with the current data. Overall, more investigations are urgently needed to provide further evidence and address the unsolved agendas.

## Conclusions

According to our meta-analysis, UGE decreased significantly with the deterioration of renal function in patients treated with SGLT2i.

## Data Availability Statement

The original contributions presented in the study are included in the article/**Supplementary Material**. Further inquiries can be directed to the corresponding authors.

## Author Contributions

LJ and XC conceptualized this study and designed the systematic review protocol. SH, CL, and FL performed the study selection and data extraction. CL and XC performed the statistical analyses. SH, CL, and XC prepared the outlines and wrote the manuscript. All authors contributed to the article and approved the submitted version.

## Funding

This work was supported by the Beijing Natural Science Foundation (no. 7202216) and the National Natural Science Foundation of China (no. 81970698 and no. 81970708). The funding agencies had no roles in the study design, data collection or analysis, decision to publish, or preparation of the manuscript.

## Conflict of Interest

LJ was employed by AstraZeneca, Merck, Metabasis, MSD, Novartis, Eli Lilly, Roche, Sanofi-Aventis, and Takeda for lecture presentations and for consulting.

The remaining authors declare that the research was conducted in the absence of any commercial or financial relationships that could be construed as a potential conflict of interest.

## Publisher’s Note

All claims expressed in this article are solely those of the authors and do not necessarily represent those of their affiliated organizations, or those of the publisher, the editors and the reviewers. Any product that may be evaluated in this article, or claim that may be made by its manufacturer, is not guaranteed or endorsed by the publisher.
