# Supplementary material for: The Urinary Glucose Excretion by Sodium–Glucose Cotransporter 2 Inhibitor in Patients With Different Levels of Renal Function: A Systematic Review and Meta-Analysis
Source: Front Endocrinol (Lausanne). 2022 Jan 27;12:814074. doi: 10.3389/fendo.2021.814074 (PMC8830597; doi:10.3389/fendo.2021.814074)
Supplement: Supplementary file 1 [file DataSheet_1.docx]

Supplementary Materials

Supplementary Figure legends

Figure S1. Funnel plots of analyses for 24h post-treatment UGE.

Figure S2. Funnel plots of analyses for 24h UGE delta changes.

Figure S3. Means plots for 24h post-treatment UGE in different levels of renal function.

Figure S4. Means plots for 24h UGE delta changes in different levels of renal function.

Figure S5. Post-treatment UGE in patients with different levels of renal function (take eGFR=60 mL/min/1.73 m^2^ as the cut-off value)

Figure S6. Post-treatment UGE in patients with different levels of renal function (take eGFR=30 mL/min/1.73 m^2^ as the cut-off value)

Figure S7. UGE delta changes in patients with different levels of renal function (take eGFR=60 mL/min/1.73 m^2^ as the cut-off value)

Figure S8. UGE delta changes in patients with different levels of renal function (take eGFR=30 mL/min/1.73 m^2^ as the cut-off value)

Supplementary Table legends

Table S1. The risk of bias for included trials of UGE assessment

Table S2.Description of 24h post-treatment UGE in different levels of renal function

Table S3.ANOVA tendency analysis for 24h post-treatment UGE in different levels of renal function

Table S4.Description of 24h UGE delta changes in different levels of renal function

Table S5.ANOVA tendency analysis for 24h UGE delta changes in different levels of renal function

Figure S1. Funnel plots of analyses for 24h post-treatment UGE.


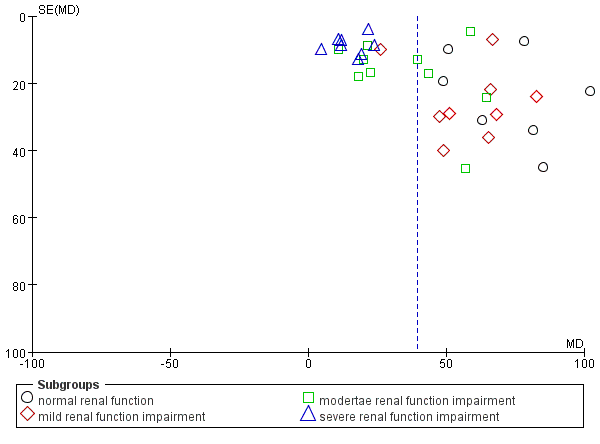


Figure S2. Funnel plots of analyses for 24h UGE delta changes.


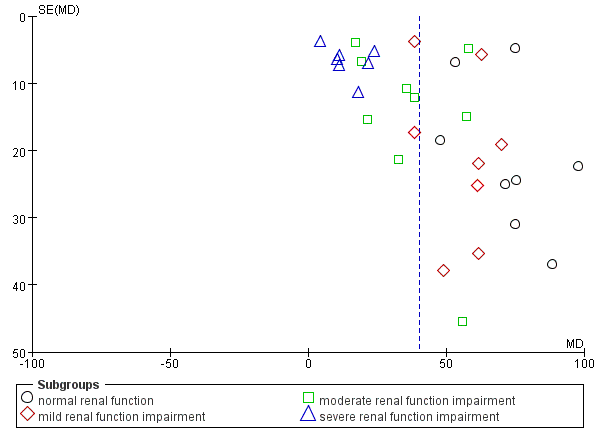


Figure S3. Means plots for 24h post-treatment UGE in different levels of renal function.

Figure S4. Means plots for 24h UGE delta changes in different levels of renal function.

Figure S5. Post-treatment UGE in patients with different levels of renal function

(take eGFR=60 mL/min/1.73 m^2^ as the cut-off value)


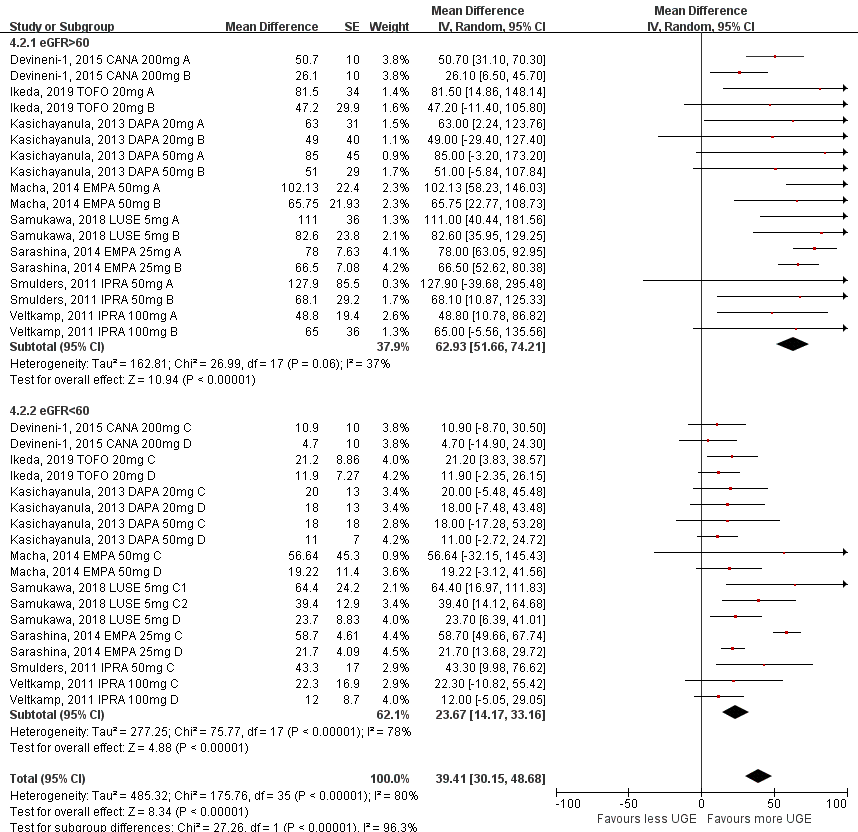


Figure S6. Post-treatment UGE in patients with different levels of renal function

(take eGFR=30 mL/min/1.73 m^2^ as the cut-off value)


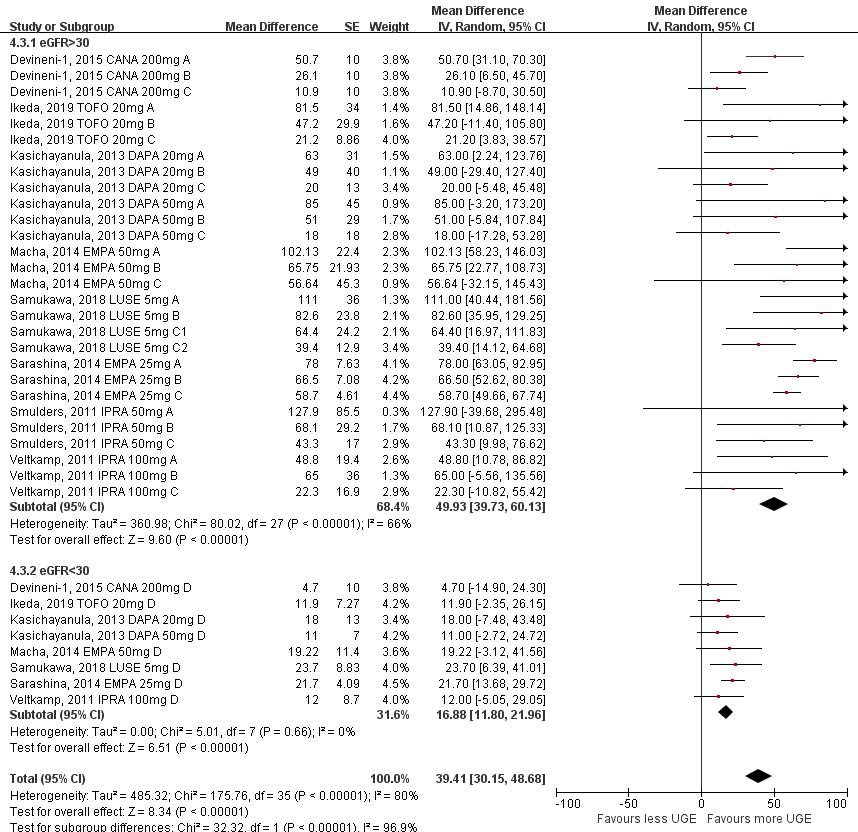


Figure S7. UGE delta changes in patients with different levels of renal function

(take eGFR=60 mL/min/1.73 m^2^ as the cut-off value)


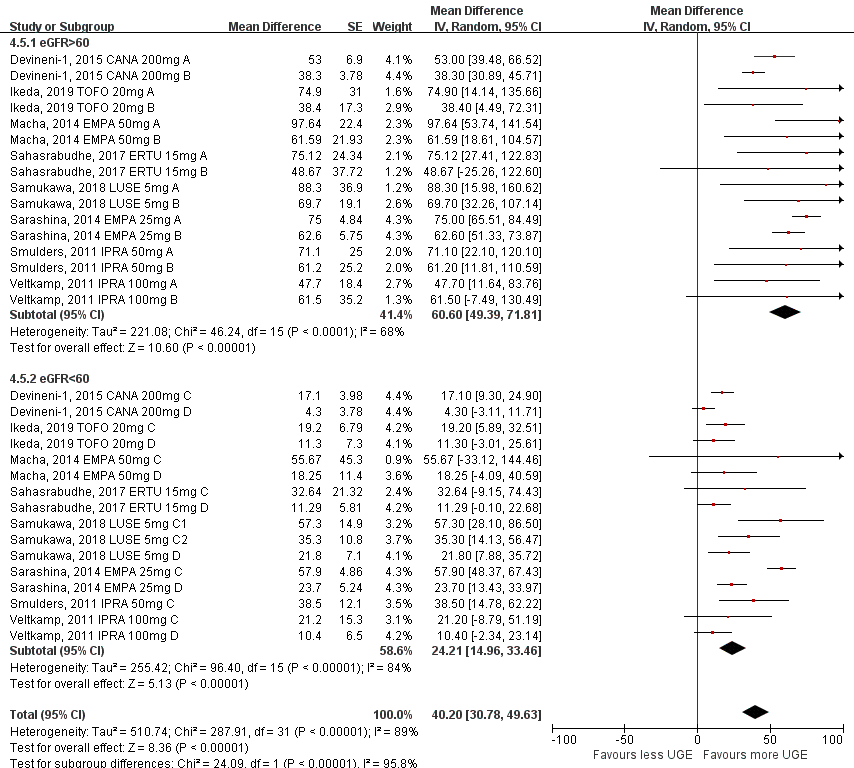


Figure S8. UGE delta changes in patients with different levels of renal function

(take eGFR=30 mL/min/1.73 m^2^ as the cut-off value)


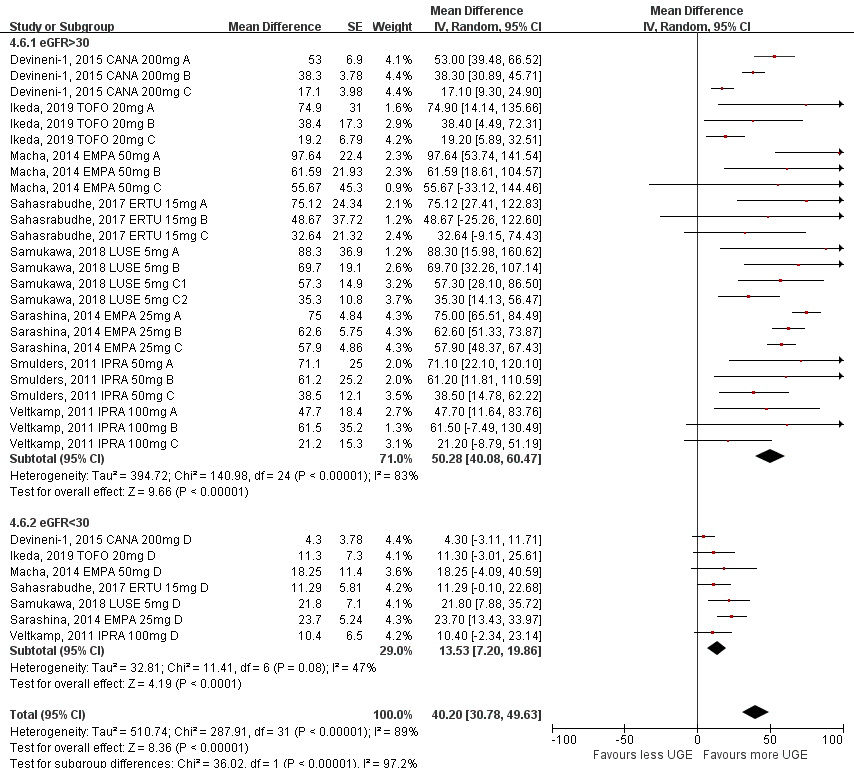


Table S1. The risk of bias for included trials of UGE assessment

| Author, year | Adequate randomization sequence generation | Adequate  allocation concealment | Blinding  of participants and caregivers | Binding of  outcome assessors  and adjudicators | Free of infrequent  missing outcome data | Free of selective outcome reporting | Free of other bias |
| --- | --- | --- | --- | --- | --- | --- | --- |
| Devineni et al, 2015^2^ | **No**  Open-label, parallel-group | **No**  Open-label, parallel-group | **No**  Open-label, parallel-group | **No**  Open-label, parallel-group | **Definitely Yes** | **Definitely yes** | **Probably yes**  Generally balanced baseline  characteristics across groups |
| Ikeda et al, 2019^3^ | **No**  Open-label, parallel-group | **No**  Open-label, parallel-group | **No**  Open-label, parallel-group | **No**  Open-label, parallel-group | **Definitely Yes** | **Definitely yes** | **Probably yes**  Generally balanced baseline  characteristics across groups |
| Macha et al, 2015^4^ | **No**  Open-label, parallel-group | **No**  Open-label, parallel-group | **No**  Open-label, parallel-group | **No**  Open-label, parallel-group | **Definitely Yes** | **Definitely yes** | **Probably yes**  Generally balanced baseline  characteristics across groups |
| Sahasrabudhe et al, 2017^5^ | **No**  Open-label, parallel-group | **No**  Open-label, parallel-group | **No**  Open-label, parallel-group | **No**  Open-label, parallel-group | **Definitely Yes** | **Definitely yes** | **Probably yes**  Generally balanced baseline  characteristics across groups |
| Samukawa et al, 2018^6^ | **No**  Open-label, parallel-group | **No**  Open-label, parallel-group | **No**  Open-label, parallel-group | **No**  Open-label, parallel-group | **Definitely Yes** | **Definitely yes** | **Probably yes**  Generally balanced baseline  characteristics across groups |
| Sarashina et al, 2014^7^ | **No**  Open-label, parallel-group | **No**  Open-label, parallel-group | **No**  Open-label, parallel-group | **No**  Open-label, parallel-group | **Definitely Yes** | **Definitely yes** | **Probably yes**  Generally balanced baseline  characteristics across groups |
| Smulders et al, 2011^8,9^ | **No**  Open-label, parallel-group | **No**  Open-label, parallel-group | **No**  Open-label, parallel-group | **No**  Open-label, parallel-group | **Definitely Yes** | **Definitely yes** | **Probably yes**  Generally balanced baseline  characteristics across groups |
| Veltkamp et al, 2011^9,10^ | **No**  Open-label, parallel-group | **No**  Open-label, parallel-group | **No**  Open-label, parallel-group | **No**  Open-label, parallel-group | **Definitely Yes** | **Definitely yes** | **Probably yes**  Generally balanced baseline  characteristics across groups |

| Table S2.Description of 24h post-treatment UGE in different levels of renal function | | | | | | | | |
| --- | --- | --- | --- | --- | --- | --- | --- | --- |
|  | N | Mean | SD | SE | 95% CIs of Mean | | Min | Max |
|  |  |  |  |  | LL | UL |  |  |
| 1 | 9 | 83.114444 | 26.9490933 | 8.9830311 | 62.399538 | 103.829351 | 48.8000 | 127.9000 |
| 2 | 9 | 57.916667 | 16.4037724 | 5.4679241 | 45.307611 | 70.525722 | 26.1000 | 82.6000 |
| 3 | 10 | 35.484000 | 19.5129218 | 6.1705277 | 21.525297 | 49.442703 | 10.9000 | 64.4000 |
| 4 | 8 | 15.277500 | 6.4083913 | 2.2657085 | 9.919951 | 20.635049 | 4.7000 | 23.7000 |
| Total | 36 | 48.509444 | 31.0678953 | 5.1779826 | 37.997581 | 59.021308 | 4.7000 | 127.9000 |

SD standard deviation, SE standard error, CI confidence intervals, LL lower limit, UL upper limit, Min minimum, Max maximum.

| Table S3.ANOVA tendency analysis for 24h post-treatment UGE in different levels of renal function | | | | | | | |
| --- | --- | --- | --- | --- | --- | --- | --- |
|  | | | SS | DF | MS | F | P |
| SSB |  | | 22105.536 | 3 | 7368.512 | 20.193 | .000 |
|  | LT | unweighted | 21852.756 | 1 | 21852.756 | 59.886 | .000 |
|  |  | weighted | 22049.781 | 1 | 22049.781 | 60.426 | .000 |
|  |  | variance | 55.754 | 2 | 27.877 | .076 | .927 |
| SSW | | | 11676.958 | 32 | 364.905 |  |  |
| Total | | | 33782.494 | 35 |  |  |  |

SS sum of squares, DF degree of freedom, MS mean square, SSB sum of squares between groups, SSW sum of squares

within groups, LLA Linear Term

| Table S4.Description of 24h UGE delta changes in different levels of renal function | | | | | | | | |
| --- | --- | --- | --- | --- | --- | --- | --- | --- |
|  | N | Mean | SD | SE | 95% CIs of Mean | | Min | Max |
|  |  |  |  |  | LL | UL |  |  |
| 1 | 8 | 72.845000 | 16.4613478 | 5.8199653 | 59.082969 | 86.607031 | 47.7000 | 97.6400 |
| 2 | 8 | 55.245000 | 11.9044217 | 4.2088487 | 45.292654 | 65.197346 | 38.3000 | 69.7000 |
| 3 | 9 | 37.201111 | 16.5217451 | 5.5072484 | 24.501374 | 49.900849 | 17.1000 | 57.9000 |
| 4 | 7 | 14.434286 | 6.9923336 | 2.6428537 | 7.967456 | 20.901116 | 4.3000 | 23.7000 |
| Total | 32 | 45.642813 | 25.0691903 | 4.4316486 | 36.604406 | 54.681219 | 4.3000 | 97.6400 |

SD standard deviation, SE standard error, CI confidence intervals, LL lower limit, UL upper limit, Min minimum, Max maximum.

| Table S5.ANOVA tendency analysis for 24h UGE delta changes in different levels of renal function | | | | | | | |
| --- | --- | --- | --- | --- | --- | --- | --- |
|  | | | SS | DF | MS | F | P |
| SSB |  | | 14116.454 | 3 | 4705.485 | 24.554 | .000 |
|  | LT | unweighted | 14113.369 | 1 | 14113.369 | 73.645 | .000 |
|  |  | weighted | 14054.928 | 1 | 14054.928 | 73.340 | .000 |
|  |  | variance | 61.526 | 2 | 30.763 | .161 | .852 |
| SSW | | | 5365.939 | 28 | 191.641 |  |  |
| Total | | | 19482.393 | 31 |  |  |  |

SS sum of squares, DF degree of freedom, MS mean square, SSB sum of squares between groups, SSW sum of squares

within groups, LLA Linear Term.

References in supplement

1. Kasichayanula S, Liu X, Pe Benito M, et al. The influence of kidney function on dapagliflozin exposure, metabolism and pharmacodynamics in healthy subjects and in patients with type 2 diabetes mellitus. Br J Clin Pharmacol. 2013;76(3):432-44.

2. Devineni D, Curtin CR, Marbury TC, et al. Effect of hepatic or renal impairment on the pharmacokinetics of canagliflozin, a sodium glucose co-transporter 2 inhibitor. Clin Ther. 2015;37(3):610-628.

3. Ikeda S, Takano Y, Schwab D, et al. Effect of Renal Impairment on the Pharmacokinetics and Pharmacodynamics of Tofogliflozin (A SELECTIVE SGLT2 Inhibitor) in Patients with Type 2 Diabetes Mellitus. Drug Res (Stuttg). 2019;69(6):314-322.

4. Macha S, Mattheus M, Halabi A, et al. Pharmacokinetics, pharmacodynamics and safety of empagliflozin, a sodium glucose cotransporter 2 (SGLT2) inhibitor, in subjects with renal impairment. Diabetes Obes Metab. 2014;16(3):215-22.

5. Sahasrabudhe V, Terra SG, Hickman A, et al. The Effect of Renal Impairment on the Pharmacokinetics and Pharmacodynamics of Ertugliflozin in Subjects With Type 2 Diabetes Mellitus. J Clin Pharmacol. 2017;57(11):1432-1443.

6. Samukawa Y, Haneda M, Seino Y, et al. Pharmacokinetics and Pharmacodynamics of Luseogliflozin, a Selective SGLT2 Inhibitor, in Japanese Patients With Type 2 Diabetes With Mild to Severe Renal Impairment. Clin Pharmacol Drug Dev. 2018;7(8):820-828.

7. Sarashina A, Ueki K, Sasaki T, et al. Effect of renal impairment on the pharmacokinetics, pharmacodynamics, and safety of empagliflozin, a sodium glucose cotransporter 2 inhibitor, in Japanese patients with type 2 diabetes mellitus. Clin Ther. 2014;36(11):1606-1615.

8. Smulders R, Ishikawa H, Nakajo I, et al. The effect of renal impairment on the pharmacokinetics and urinary glucose excretion of the SGLT2 inhibitor ipragliflozin (ASP1941) in Japanese type 2 diabetes mellitus patients. Poster presentation at the 47th Annual Meeting of the European Association for the Study of Diabetes (EASD), Lisbon, Portugal; September 12–16, 2011. Abstract Number: 847.

9. Kadokura T, Zhang W, Krauwinkel W, et al. Clinical pharmacokinetics and pharmacodynamics of the novel SGLT2 inhibitor ipragliflozin. Clin Pharmacokinet. 2014;53(11):975-88.
